# The characteristic of otoacoustic emissions in full-term neonates according to ABO blood groups^☆^

**DOI:** 10.1016/j.bjorl.2019.10.012

**Published:** 2019-12-10

**Authors:** Aifeng Li, Guoqiang Gao, Ningyu Wang, Tao Fu, Fugao Zhu, Xiaoheng Zhang, Jie Liu

**Affiliations:** a The Affiliated Hospital of Qingdao University, Department of Otolaryngology Head and Neck Surgery, Qingdao, China; b The Affiliated Hospital of Qingdao University, Department of Obstetrics, China; c Capital Medical University, Beijing Chaoyang Hospital, Department of Otorhinolaryngology Head and Neck Surgery, China

**Keywords:** ABO blood-group system, Spontaneous otoacoustic emissions, Distortion product otoacoustic emissions, Noise-induced hearing loss, Sistema ABO de grupos sanguíneos, Emissões otoacústicas espontâneas, Emissões otoacústicas por produto de distorção, Perda auditiva induzida por ruído

## Abstract

**Introduction:**

Previous research has suggested that individuals with different blood groups show varied incidences of noise-induced hearing loss. The reduced otoacoustic emissions amplitudes indicate the higher possibilities of outer hair cell damage for noise exposure.

**Objective:**

The objective is to analyze the characteristics of otoacoustic emissions, including the occurrence of spontaneous otoacoustic emission and the amplitudes of distortion product otoacoustic emission at certain frequencies in full term neonates with different ABO blood groups.

**Methods:**

A total of 80 selected full-term female neonates who passed the initial newborn hearing screen were enrolled into the study, with equal number of participants in four ABO blood groups (Blood Group A, Blood Group B, Blood Group AB, Blood Group O). Measurements of spontaneous otoacoustic emission and distortion product otoacoustic emission were performed in both ears for all participants.

**Results:**

(1) The blood group O participants showed significantly fewer spontaneous otoacoustic emission occurrences than the other three blood groups (A = 70%, B = 80%, AB = 67%, O = 25%, *p* <  0.05). (2) The blood group O participants showed lower DPOAE amplitudes at 1257 Hz (M = 4.55 dB, SD = 8.36), 1587 Hz (M = 11.60 dB, SD = 6.57), 3174 Hz (M = 7.25 dB, SD = 5.99), 5042 Hz (M = 13.60, SD = 6.70) than participants with the other three blood groups in left ears (*p* < 0.05). In right ears, the blood group O participants showed reduced amplitudes at 1257 Hz (M = 6.55 dB, SD = 8.36), 1587 Hz (M = 13.60 dB, SD = 6.57), 3174 Hz (M = 7.65 dB, SD = 6.43), 5042 Hz (M = 13.65 dB, SD = 6.50) than participants from non-O blood groups (*p* < 0.05).

**Conclusion:**

Female individuals with blood group O have lower otoacoustic emissions values than individuals with the other three blood groups. We need to further investigate the possible relationships between ABO blood group and cochlear function, including the potential influences of noise damage on cochlear outer hair cells.

## Resumo

**Introdução:**

Pesquisas anteriores sugeriram que indivíduos de diferentes grupos sanguíneos apresentam incidências distintas de perda auditiva induzida por ruído. As amplitudes reduzidas das emissões otoacústicas indicaram maiores ou menores possibilidades de danos às células ciliadas por exposição a ruídos.

**Objetivo:**

O objetivo do estudo foi analisar as características das emissões otoacústicas, incluindo a ocorrência de emissões otoacústicas espontâneas e as amplitudes de emissões otoacústicas por produto de distorção em determinadas frequências em neonatos a termo de diferentes grupos sanguíneos do sistema ABO.

**Método:**

Foram incluídos 80 neonatos a termo selecionados na triagem auditiva neonatal inicial para participar do estudo, com número igual de participantes de grupos sanguíneos do sistema ABO (grupo sanguíneo A, grupo sanguíneo B, grupo sanguíneo AB e grupo sanguíneo O). As emissões otoacústicas espontâneas e emissões otoacústicas por produto de distorção foram medidas em ambas as orelhas de todos os participantes.

**Resultados:**

(1) Os participantes do grupo sanguíneo O apresentaram ocorrências de emissões otoacústicas espontâneas significantemente menores do que os dos outros três grupos sanguíneos (A = 70%, B = 80%, AB = 67%, O = 25%,*p* <  0,05). (2) Os participantes do grupo sanguíneo O apresentaram amplitudes de emissões otoacústicas por produto de distorção mais baixas a 1257 Hz (M = 4,55 dB, DP = 8,36), 1587 Hz (M = 11,60 dB, DP = 6,57), 3174 Hz (M = 7,25 dB, DP = 5,99), 5042 Hz (M = 13,0, DP = 6,70) do que os participantes dos outros três grupos sanguíneos nas orelhas esquerdas (*p* <  0,05). Nas orelhas direitas, os participantes do grupo sanguíneo O apresentaram amplitudes reduzidas em 1257 Hz (M = 6,55 dB, DP = 8,36), 1587 Hz (M = 13,60 dB, DP = 6,57), 3174 Hz (M = 7,65 dB, DP = 6,43), 5042 Hz (M = 13,65 dB, DP = 6,50) em comparação aos participantes de grupos sanguíneos não O (*p* <  0,05).

**Conclusão:**

Os indivíduos do sexo feminino do grupo sanguíneo O apresentaram valores menores de emissões otoacústicas do que os indivíduos dos outros três grupos sanguíneos. É necessário continuar a investigar as possíveis relações entre o grupo sanguíneo ABO e a função coclear, inclusive as possíveis influências do dano por ruídos às células ciliadas externas da cóclea.

## Introduction

Otoacoustic emissions (OAEs) are low level sounds generated from the cochlea without any external stimulation. OAEs are an objective indication of normal cochlear function.^1^ It can be further classified into transiently evoked otoacoustic emission (TEOAE), spontaneous otoacoustic emission (SOAE) and distortion product otoacoustic emission (DPOAE).

SOAE is widely recognized as the product of self-sustained oscillations of Outer Hair Cells (OHC).^2^ It has been suggested that it may be influenced by many factors including middle ear status, age and gender.^2^ The prevalence rate of SOAE is significantly higher in female than in male Chinese newborns. The frequencies of SOAE in newborns appeared to be higher than those reported in normal hearing adults. At the cochlear level, most studies reported asymmetries between right and left ears, manifested in a higher occurrence of SOAE in right ears.^3^

OHC are typically the first section of the auditory system to be significantly influenced by exposure to certain environments, such as noise.^4^ Therefore, changes in OAE amplitude can reflect early injuries of the cochlear OHC. Hall^5^ reported that OAE testing was twice as sensitive as pure-tone audiometry in detecting changes in the hearing threshold levels and suggested that it could improve monitoring for Noise-Induced Hearing Loss (NIHL) in the workplace. A previous study^6^ also indicated that OAE could reflect noise-induced changes in cochlear OHC, which was undetected by pure-tone audiometric tests. OAE might therefore act as superior diagnostic predictor for NIHL. Abnormally low OAE response amplitudes may also suggest greater risk of developing NIHL.

The blood groups are divided into four groups: A, B, O and AB, based on the different blood group antigens on the surface of red blood cells. Different blood groups can have varied susceptibility to certain diseases, due to differential genetic expressions of antigens on each blood group. A study conducted by Nair^6^ suggested that air force personnel with blood Group O had a higher incidence of NIHL compared to personnel with other blood groups, which was consistent with other published studies. Dogru^7^ found a potential correlation between ABO blood groups and NIHL in a study of 176 workers, where participants with blood Group O had higher incidences of NIHL than non-O blood group participants. These studies suggest that the incidence of NIHL has a potential relationship with the blood groups. Blood groups could thus be a predictor of the development of NIHL.

Another previous study hypothesized that there were significantly different OAEs values among the four blood groups in normal hearing individuals.^8^ Non-O blood group individuals demonstrated higher OAE prevalence in SOAE and larger DPOAE amplitudes at certain frequencies than blood Group O individuals. However, the different performances of OAE among the four blood groups in neonates are not clear, especially during the first month. It is also worthwhile to further investigate the relationship between different OAE amplitude and the hematological risk factor for NIHL.

Therefore, the aims of this study are to determine (1) the incidence of SOAE among the four blood groups in neonates; (2) DPOAE response amplitudes at selected frequencies among the four blood groups in neonates and (3) whether there is a significant difference in OAE recordings between left and right ears.

## Methods

### Participants

80 selected full-term female neonates were enrolled into the study from local hospital between January 2018 and December 2018. Because previous studies^9^ have shown significant gender differences of SOAE pass rates in normal full-term neonates, the participants included in the study were all of the same gender. All participants included in the study fulfilled the following criteria: they were ethnic Han Chinese; had accurate knowledge of their blood groups from blood tests. There were 20 participants representing each of blood groups A, AB, B, and O respectively. Because the population of Rh- negative blood type in ethnic Han is present in 0.9 %, participants were assumed to have an Rh-positive blood type.^10^ Their gestational ages at birth ranged from 38 to 40 weeks. Their testing age were 7.3 ± 3.5 days in blood Group O; 7.85 ± 3.4 days in blood Group A; 7.5 ± 2.5 days in blood Group B; 6.75 ± 3.6 days in blood Group AB, and there was no statistic difference among four blood groups (*p* = 0.765). There was no history of auditory pathology in the family members of any participant. They had no history of ear infections or major health problems such as congenital hypothyroidism. In order to be included in the study, all subjects had passed the initial newborn hear screening test. They had recorded middle ear function, such as static admittance, equivalent ear-canal volume, tympanic width and tympanic peak pressure in both ears after cleaning the external canal. The parents of each neonate were provided with written informed consent prior to entry into the study. The research study was approved by the Human Research Ethics Committee and written informed consent was obtained from each participant before assessment (Research Number：QYFYWZLL25542).

### Design and procedures

All auditory tests and records were performed in a sound booth (<30 dB) (A) while the neonates were quiet or sleeping to obtain a more precise and reliable result. Any recording process that was disrupted, especially if the probes disturbed or unsealed by the movement of the neonates, was repeated after adjusting the probe again in the outer ear canal.

Tympanometry was performed in both ears using a screening middle ear analyzer (the Interacoustics AA220, Assens, Denmark) with appropriate calibration. Tympanograms of both ears were recorded in each participant using a 1000 Hz probe tone. According to the Baldwin classification method, positive peak suggested normal middle ear function.^11^

As in Chen’s research,^12^ recordings of SOAE and DPOAE were conducted using the Capella OAE (Madsen, Denmark). An appropriate size silicon rubber measurement probe tip was inserted in right and left ear canals in randomized order for OAE measurement.

SOAE test: Five hundred epochs of 80 ms after a synchronizing click stimulus (duration 1–1.5 ms, intensity of 70–80 dBSPL) were acquired (sampling frequency 25 kHz). Each SOAE recording needed to meet the two following criteria: the spectral line (above 500 Hz) exceeded the average of all other spectral peaks within 40 Hz by 3 dB or more and the absolute amplitude of the signal exceeded 25 Dbspl.^13^

DPOAE test: To obtain robust responses, two pure-tone stimuli at 65/50 dBSPL (f2:f1 = 1.22) were presented to the ear simultaneously. A total of four f2 frequencies spaced at one-third octave intervals were used 1257 Hz, 1587 Hz, 3174 Hz, 5042 Hz.

### Statistical methods

Statistical analysis was carried out with the use of the SPSS software (V.17.0; SPSS Inc., USA). The occurrences of SOAE among the participants with the different blood groups and the average amplitudes of DPOAE with standard deviations were analyzed by the descriptive statistics. Inferential statistic analysis was used for investigating the possible differences among four blood groups about DPOAE amplitudes. Chi-square tests were used to detect differences in SOAE prevalence in terms of numbers of ears with four blood groups. One-way ANOVA test evaluated possible differences in DPOAE amplitudes at testing frequencies among four blood groups. The post hoc pair wise comparison was used to ascertain significant results. The selected level of significance was *p* < 0.05.

## Results

Overall, a total of 97/160 years in the 80 female neonates were tested for SOAE. Bilateral SOAE testing was not possible in all babies because during the test, some neonates began to cry or feel hungry. In such situations, their mothers or the baby's guardians did not allow the test to be continued and the data were not collected from each ear.

### SOAE prevalence in full-term neonate

Table 1 showed the prevalence of SOAE among four blood groups. A total of 97 out of 160 ears (60.63 %) displayed SOAE. A Chi-square test demonstrated significant difference among the four blood groups X^2^ (3, n = 160) = 11.58, *p* = 0.009.

**Table 1:** SOAE prevalence in full-term neonates.

Group	Number of testing ears	Number of SOAE ears	Prevalence rate (%)	Chi-square test		
				X^2^	*p*	
A	40	28	70	11.58		0.009
B	40	32	80			
AB	40	27	67.5			
O	40	10	25			
Total	160	97	60.63			

Post-hoc tests showed significant differences among four blood groups with *p* = 0.05. The subjects in blood Group O had significantly fewer occurrences of SOAE than subjects in blood Group A (X^2^ [1, n = 80] = 5.12, *p* = 0.024) and blood Group B (X^2^ [1, n = 80] = 10.453, *p* = 0.001), also blood Group AB (X^2^ [1, n = 80] = 4.114, *p* = 0.043). There was no significant difference in other three blood groups. SOAE prevalence in ear side demonstrated significant difference (Table 2). The blood Group O subjects demonstrated the fewest number of SOAE tested in right ears. There was a significant difference in the ratio among the blood groups (X^2^ [80, n = 3] = 19.72, *p* =  0.001). The blood Group O subjects demonstrated the fewest number of SOAE tested in left ears. There was a significant difference in the ratio among the blood groups (X^2^ [80, n = 3] = 11.83, *p* =  0.008). The occurrence of SOAE in right ears and left ears had significant difference (X^2^ [160, n = 1] = 4.425, *p* = 0.035).

**Table 2:** Ear side of SOAE prevalence in full-term neonates.

	Blood group A	Blood group B	Blood group AB	Blood group O	Chi-square test	
					X^2^	*p*
Left ear					11.83	0.008
Number of testing ears	20	20	20	20		
Number of SOAE ears	12	14	12	4		
Prevalence rate (%)	60	70	60	20		
Right ear					19.72	0.001
Number of testing ears	20	20	20	20		
Number of SOAE ears	16	18	15	6		
Prevalence rate (%)	80	90	75	30		

### DPOAE findings in four blood groups

Table 3 showed the threshold data of DPOAE at four frequencies among four blood groups both in left and right ears and there was no statistical difference across all the frequencies. A one-way ANOVA test showed significant difference of DPOAE amplitudes at selected frequencies both in right ears and left ears. In left ears, statistic analysis showed significant differences on amplitudes of DPOAE at 1257 Hz (F[3.76] = 9.396, *p*  = 0.001) (Table 4); at 1587 Hz (F[3.76] = 10.247, *p* =  0.002); at 3174 Hz (F[3.76]  = 14.273, *p* =  0.001); at 5042 Hz (F[3.76] = 10.610, *p* = 0.001).

**Table 3:** DPOAE threshold at four frequencies in left and right ear among four blood group neonates.

Blood group	Threshold at 1257 Hz (dB)	Threshold at 1587 Hz (dB)	Threshold at 3174 Hz (dB)	Threshold at 5042 Hz (dB)	One-way ANOVA	
					F	*p*
Left ear						
O type	18.75 ± 3.00	19.15 ± 3.08	19.05 ± 3.02	20.00 ± 2.99	0.628	0.599
A type	19.95 ± 3.20	19.00 ± 3.01	19.40 ± 2.93	20.45 ± 3.03	0.866	0.462
B type	19.55 ± 3.03	19.50 ± 3.01	19.00 ± 2.95	18.50 ± 3.46	0.557	0.645
AB type	18.60 ± 3.05	20.20 ± 3.32	19.90 ± 3.18	19.35 ± 2.85	1.025	0.386
Right ear						
O type	19.35 ± 2.82	19.20 ± 3.17	18.80 ± 3.00	19.15 ± 2.90	0.112	0.947
A type	19.20 ± 2.73	19.25 ± 2.80	20.40 ± 3.15	18.65 ± 3.03	1.256	0.295
B type	18.85 ± 3.12	19.05 ± 2.86	19.35 ± 2.93	20.00 ± 2.99	0.571	0.636
AB type	18.80 ± 3.21	18.80 ± 3.00	19.00 ± 2.81	19.80 ± 2.76	0.521	0.669

**Table 4:** DPOAE amplitudes at four frequencies in left and right ear among four blood group neonates.

DPOAE frequency	Amplitude of blood group O (dB)	Amplitude of blood group A (dB)	Amplitude of blood group B (dB)	Amplitude of blood group AB (dB)	One-way ANOVA	
					F	*p*
Left ear						
1257 Hz	4.55 ± 8.36	8.20 ± 8.20	16.85 ± 7.24	13.00 ± 7.60	9.396	0.001
1587 Hz	11.60 ± 6.57	17.95 ± 6.53	21.30 ± 4.68	19.10 ± 5.26	10.247	0.002
3174 Hz	7.25 ± 5.99	13.45 ± 2.82	15.25 ± 2.69	13.30 ± 4.13	14.273	0.001
5042 Hz	13.60 ± 6.70	19.95 ± 6.24	23.30 ± 4.38	21.10 ± 5.28	10.610	0.001
Right ear						
1257 Hz	6.55 ± 8.36	10.20 ± 8.20	18.85 ± 7.24	15.10 ± 7.64	9.428	0.002
1587 Hz	13.60 ± 6.57	19.95 ± 6.53	23.30 ± 4.68	21.10 ± 5.26	10.247	0.001
3174 Hz	7.65 ± 6.43	14.25 ± 2.77	16.25 ± 2.69	14.25 ± 2.57	17.910	0.001
5042 Hz	13.65 ± 6.50	19.90 ± 6.26	23.25 ± 4.39	21.10 ± 5.24	10.617	0.001

**Figure 1:**
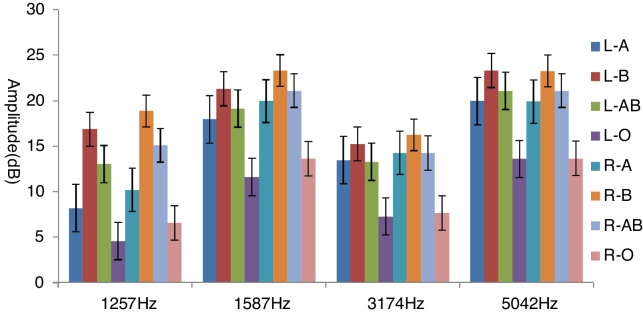
The bar chart showed DPOAE amplitudes at 1257 Hz, 1587 Hz, 3174 Hz and 5042 Hz in Left ears (L) and Right ears (R) among four blood groups. The x axis displayed the DPOAE frequencies (Hz), the y axis displayed the mean amplitude (dB) evoked among four blood group subjects. Vertical error lines stand for standard error.

Post-hoc comparison showed that the blood group O subjects had lower DPOAE amplitudes at 1257 Hz (M =4.55 dB, SD = 8.36), 1587 Hz (M =11.60 dB, SD = 6.57), 3174 Hz (M =7.25 dB, SD = 5.99) and 5042 Hz (M = 13.60 dB, SD = 6.70) than subjects with other three blood groups in left ears (*p* < 0.05). There was no significant difference among other three blood groups (Fig. 1).

In right ears, statistic analysis showed significant differences on amplitudes of DPOAE at 1257 Hz (F [3.76] = 9.428, *p* = 0.002) (Table 4); at 1587 Hz (F[3.76] = 10.247, *p* =  0.001), at 3174Hz (F[3.76] = 17.910, *p*  = 0.001), at 5042 Hz (F[3.76] = 10.617, *p* =  0.001). In right ears, post-hoc comparison showed the blood Group O participants had reduced amplitudes at 1257 Hz (M = 6.55 dB, SD = 8.36), 1587 Hz (M = 13.60 dB, SD = 6.57), 3174 Hz (M = 7.65 dB, SD = 6.43), 5042 Hz (M = 13.65 dB, M = 6.50) than participants from A, B and AB groups (*p* <  0.05). No significant differences were found among other three blood groups (Fig. 1).

## Discussion

The purpose of this research was to verify the statistical difference in SOAE incidence and DPOAE amplitude among the four blood groups in neonates. We carefully screened for gender, age, ethnicity, hearing level, physical condition and test environment when conducting analysis of the subjects. For the 80 female subjects, results showed that the subjects with blood group O had the lowest SOAE incidence among the four blood groups in both ears. Also, subjects with blood group O revealed lower DPOAE amplitudes at 1257 Hz, 1587 Hz, 3174 Hz and 5042 Hz than those from the other three blood groups, both in right and left ears. OAE are thought to represent the normal functioning of the active feedback mechanism in the cochlea.^14^ The function of OHC begins before 30 weeks of gestation. After its functional onset in human, OHC undergos maturational changes over a period of several weeks.^15^ Since OAE comes from OHC of the cochlea, the differences in OAE amplitude among female subjects also reflect potential decreased OHC activities.

The present study found that blood Group O subjects had lower SOAE incidence than the other three blood groups, while DPOAE amplitudes at 1257 Hz, 1587 Hz, 3174 Hz and 5042 Hz were also found to be lower than other three blood groups. It can be inferred that the function of OHC in blood Group O is less active than other three blood groups. According to these results, we suggest that individuals with blood Group O may be more susceptible to NIHL than individuals with a non-O blood group. The results of this present research are consistent with previous research conducted by Dogru.^7^ Dogru studied that NIHL was found in 23 persons with blood Group A (32.0 %), 35 with blood Group O (58.3 %), 10 with blood Group B (38.5 %) and 7 with blood Group AB (38.9 %), respectively. People with blood Group O are more susceptible to NIHL than those with non-O blood groups.

A previous study^12^ discovered that blood group O participants exhibited significantly lower SOAE prevalence (3.3 %) and reduced amplitudes of DPOAE on average than participants with a non-O blood group. The blood Group O population has genetic susceptibility to factors such as larger ear canal volume, ossicular chain mass, or a more active cochlear efferent system,^16^ which affect the performance of OAE. The large external auditory canal volume weakens OAE signals, which is not conducive to the detection device that records the OAE. There is a close correlation between the regulation of SOAE and the cochlear efferent system. Due to medial olivocochlear system dysfunction, the reduced inhibitory function leads to a higher occurrence and larger amplitudes of OAE.^17^ An alternative explanation for the asymmetries in OAE could be the difference in strength of the efferent system influence by the medial olivocochlear system on the OHC.^18^ The different prevalence in ear-side might also depend on the asymmetries of the efferent auditory system which led to the asymmetries of hearing sensitivity.^19^, ^20^

The present study indicated that SOAE occurrence and DPOAE amplitudes at the selected frequency for blood Group O were statistically different from the other three blood groups in neonates. This suggests that people with blood Group O contain certain genetic predisposing factors, which may influence the imperfect growth of cochlear OHC and have an effect on the performances of OAE. Human blood group antigens are transiently expressed in developing cochlear hair cells. This temporal antigen expression seems to correspond to the main events of inner ear division (e.g., hair cell development, synaptogenesis, ciliogenesis).^21^ Previous studies^12^ have shown that the number of OHC in the blood group O population are fewer than in the non-O blood group, and the function of OHC are less active. It may be possible that the blood group O population is more prone to spontaneous hair cell loss at an earlier age.^22^ Biological differences between blood groups can provide evidence to explain the disadvantages of blood group O individuals in auditory physiology. Individuals with blood group O lack histo-blood Group A transferase and histo-blood Group B transferase while individuals with a non-O blood group contain A and B transferase.^23^ A lack of these functional proteins can have potentially negative effects on the cochlear function of the blood Group O population. Also, the concentration of vonWille brand factor (vWF) in the blood Group O system is lower than that of non-O blood groups.^24^ vWF is a carrier protein for factor VIII, which prevents proteolysis of factor VIII. Blood Group O individuals have reduced clotting factors in their blood, which may have played an important role in rapid biological tissue repair mechanisms. For example, when the fine tissues are damaged, they will coagulate and avoid aggravated damage.

Although hearing sensitivity is at a normal range, the OHC are prone to subtle damage due to severe vibration of the basement and tectorial membranes, when OHC are exposed to noise environments. At the same time, the blood vessels and capillaries which are responsible for supply nutrients to the cochlear are also damaged. Because the blood coagulation process in blood group O individuals is not as fast and effective as in non-O blood group, it cannot inhibit further damage to tissues that can be damaged at an early stage, and consequently the number of healthy OHC is further reduced. A better understanding of the physiological function of blood group antigen associated with OHC development could help explain the auditory inferiority of blood group O individuals.

The findings suggest that an ABO blood group is a risk factor for development of hearing loss, increasing the susceptibility to NIHL. Although the responses may be significantly lower, those could still be adequate for newborns to pass the screening test. This suggests that the loss of OHCs in individuals with blood Group O may not be large enough to cause an elevation in threshold. The difference in blood groups could simply be a genetic predisposition. It is not a congenital hearing defect in newborns. Other factors such as larger ear canal volume or stronger efferent system have effects on OAE amplitude. The reduce of OHCs can be influenced separately by, or by a combination of environmental, medical (this last sentence is quite unclear to me).

In future large scale studies are clearly needed to confirm the present findings. Moreover, the measurement of nonlinear and linear TEOAE should be used to detect cochlear responses. SOAE and DPOAE amplitudes alone cannot reflect the cochlear function completely. Apart from DPOAE amplitudes, the fine structure and frequency space of DPOAE components are also of importance to indicate cochlear status. Therefore, it would be useful to compare the amplitudes caused by each DPOAE component in future studies.

## Conclusions

The current research showed that the female subjects with blood Group O had significantly fewer occurrence of SOAE, and significantly lower DPOAE amplitudes at frequencies of 1257 Hz, 1587 Hz, 3174 Hz and 5042 Hz both in left and right ears when compared with other three blood group subjects. The current research suggested that individuals with blood Group O might have auditory inferiority at a physiologic level and supported the view that blood Group O individuals might have higher opportunity to suffer NIHL. We need to further study the relationship between ABO blood group and auditory function, especially molecular aspects of OHC and the function of the efferent system. It can be used to assess the effect of ABO blood group on auditory sensitivity, speech comprehension and auditory processing.

## Conflicts of interest

The authors declare no conflicts of interest.
